# A Method for Reducing the Temperature Sensitivity of a Single-Base Propellant by Adding Ultra-Fine RDX Particles

**DOI:** 10.3390/polym18101156

**Published:** 2026-05-08

**Authors:** Sihan Zhu, Yingbo Wang, Qixuan Ying, Zongcheng Jiang, Ruifan Zhao, Yinan Yang, Tong Sun, Yeqin Weng, Bin Xu, Weidong He

**Affiliations:** 1 School of Chemical Engineering, Nanjing University of Science and Technology, Nanjing 210094, Chinawybchem@163.com (Y.W.);; 2 Qian Xuesen College, Nanjing University of Science and Technology, Nanjing 210094, China; 3 Key Laboratory of Special Energy Materials, Ministry of Education, Nanjing 210094, China

**Keywords:** single-base propellant, Ultra-fine RDX, temperature sensitivity coefficient, modification, combustion performance

## Abstract

The temperature sensitivity coefficient greatly affects the interior ballistic performance of propellant charges. Even under consistent loading conditions, variations in environmental temperature can lead to maximum chamber pressure fluctuations of 40–80 MPa, thereby compromising weapon efficiency and operational safety. In order to obtain a single-base propellant with a higher energy and lower temperature sensitivity coefficient, ultra-fine RDX particles were added into the single-base propellant. The difference in thermal expansion coefficients between RDX and the single-base propellant matrix leads to temperature-dependent microcracking. These microcracks increase the burning surface area at low temperatures, compensating for the reduced chemical reaction rate and thereby lowering the temperature sensitivity coefficient. A scanning electron microscope (SEM) was used to observe the inner structure of the single-base propellant with and without RDX particles. The thermal mechanical analysis (TMA) results, together with SEM observations, reveal that the interfaces between the propellant matrix and the RDX particles are temperature-dependent. As a result, the burning surface area of the modified single-base propellant varies with temperature, contributing to a reduced temperature sensitivity coefficient. Closed bomb tests were conducted to verify this inference, and the obtained curves and relevant quickness (RQ) values showed that the modified single-base propellant had stable burning behavior and lower temperature sensitivity. This study leverages the structural interactions between high-energy fillers and polymer matrices to provide a potential strategy for designing climate-resilient ammunition.

## 1. Introduction

The optimization of interior ballistic parameters is crucial for enhancing weapon power and stability [1]. For large-caliber guns especially, the accurate prediction and control of combustion behavior are essential. For a long time, ambient temperature has been one of the key factors limiting the development of firearms [2]. It is well known that the performance of firearms is significantly affected by temperature. Muzzle velocity and the maximum chamber pressure, which are two representative parameters of firearms, increase as temperature rises and vice versa. This phenomenon is mainly caused by the initial temperature of propellant charges and can be described by a parameter called the temperature sensitivity coefficient [3]. Therefore, the value of this coefficient shows the degree of dependence that propellant charge performance has on ambient temperatures.

Propellant charges are expected to have the highest performance under the allowable pressure of the artillery system and maintain their performance under any circumstance [4]. Ideally, the initial temperature of the propellant should not influence the ballistic properties of ammunition. Unfortunately, due to the presence of the temperature sensitivity coefficient, the actual propellant charge always has different performances at different temperatures. Moreover, conventional propellants usually have larger temperature sensitivity coefficients, which means that their performances will change significantly with changes in temperature. This issue also applies to solid rocket engines—the initial temperature of the propellant directly influences the engine’s thrust curve, specific impulse, and combustion chamber pressure, thereby affecting the missile’s range and flight stability [5]. If a propellant with a low temperature sensitivity coefficient is available, this situation will be greatly improved, and this propellant will help reduce the weapons’ dependence on ambient temperature. Therefore, the study of propellants with low temperature sensitivity coefficients can have valuable input in the development of firearms [6,7].

The temperature coefficient of propellants originates from two factors: the variation in the burning rate of the propellant itself and the alteration in the initial combustion surface area of the charge [8]. Therefore, addressing the temperature coefficient involves tackling these two aspects, either by modifying the burning rate of the propellant at different temperatures or by leveraging its brittleness and fragmentation properties to adjust the surface area of the charge under varying temperatures [2].

Currently, methods for reducing the temperature sensitivity coefficient of propellants are mainly classified into chemical and physical approaches. The chemical method involves introducing catalysts to produce a platform effect, thereby lowering burning rate temperature sensitivity. This approach exhibits excellent performance in rocket propellants [9,10], such as through the addition of lead salts or copper salts. However, for gun propellants with more stringent combustion conditions, no successful applications have been reported to date. The physical method adjusts the propellant’s combustion surface area by modifying its physical structure, thereby optimizing combustion performance [11,12] and achieving a lower temperature sensitivity coefficient—essentially compensating for reduced combustion reaction activity through the altered combustion surface area. Existing physical techniques include: crushing propellants to create microcracks [13]; compressing coated spheroidal propellants to achieve varying combustion surface areas across different temperatures [14,15]; embedding propellant particles in different matrices by leveraging temperature-dependent interfacial cohesion [16]; and penetrating surface holes, in which several coating agents are used to adjust the burning surface [17,18,19] and particle size distribution [20] of the propellant. While these methods have shown promise in laboratory settings, they often suffer from poor process controllability and insufficient batch-to-batch consistency. Research aimed at reducing propellant temperature sensitivity primarily follows two mainstream technical approaches: first, the use of Surface-Coated Dual-Basis (SCDB) propellant technology, which involves coating existing dual- or triple-base propellants with specific combustion regulators [21,22], and second, the development of novel low-temperature-coefficient (LTC) propellants by replacing traditional high-energy components with advanced plasticizers such as nitroglycerin or diethylene glycol dinitrate [23,24,25]. In recent years, reducing temperature sensitivity through microstructural design has emerged as a novel strategy. For instance, Chen et al. [26] ingeniously utilized the thermal expansion and contraction properties of materials in solid propellants, employing a “combustion surface compensation” mechanism to significantly lower the combustion rate temperature coefficient across broad temperature ranges. Although this structural compensation strategy originated from propellant research, its principle may serve as a reference for designing novel propellants with low temperature coefficients.

To improve energy and combustion performance, nitramine components such as RDX (cyclotrimethylenetrinitramine, the abbreviation of which is derived from research department explosive) [27] are incorporated into cellulose-based matrices to form specialized network structures [28]. Existing studies on RDX have primarily focused on its thermal decomposition kinetics and energetic characteristics [29,30], and systematic investigations regarding its effect on reducing temperature sensitivity remain limited [31]. In particular, the mechanism by which RDX particles modulate the burning surface through physical interactions with the propellant matrix, thereby influencing the temperature dependence of combustion, has not been fully clarified.

In this study, a modification strategy involving the incorporation of ultra-fine RDX particles into a single-base propellant is proposed. Taking advantage of the significant mismatch in thermal expansion coefficients between the single-base propellant matrix and RDX particles, a temperature-dependent combustion surface area is achieved. At low temperatures, differential contraction between RDX and the matrix induces interfacial microcracks (voids). These microcracks increase the effective burning area at low temperatures, thereby compensating for the decrease in the chemical reaction rate induced by low temperature. Conversely, at high temperatures, particle expansion and matrix softening lead to the closure of interfacial gaps, resulting in a reduced burning area. This self-regulation mechanism is expected to simultaneously enhance the energy level and reduce the temperature sensitivity of the propellant. The combustion performance and mechanical properties of the resulting modified single-base propellant are also investigated in this work.

## 2. Theoretical Principles of Reducing Temperature Coefficient

Equations (1)–(4), (7), (8), and (10) presented in this section are derived from Professor J. Corner’s book *Theory of the Interior Ballistics of Guns* [32]. Considering the low-temperature sensitivity and high-energy characteristics of the propellant, its combustion behavior in a sealed propellant charge is governed by Equations (5), (6) and (9). This model adheres to the computational criteria proposed by Xu et al. [33]. Equations (11) and (13)–(17) mentioned later are all referenced from Military Internal Ballistics [32].

Equations (1) and (2) are always used to describe the interior ballistic process:

(1) $$\begin{array}{c} Sp(l_{\psi }+l)=f\omega \psi -\frac{\theta }{2}\varphi mv^{2} \end{array}$$

Here,

(2) $$\begin{array}{c} l_{\psi }=l_{0}[1-\frac{\Delta }{\delta }(1-\psi )-\alpha \Delta \psi ] \end{array}$$

Differentiating Equation (1), we obtain

(3) $$\begin{array}{c} \frac{\mathrm{dp}}{\mathrm{dt}}=\frac{1}{l_{\psi }+l}\{\frac{f\omega }{S}[1+(\eta -\frac{1}{\delta })\frac{p}{f}]\frac{d\psi }{\mathrm{dt}}-\nu (1+\theta )p\} \end{array}$$

When the chamber pressure reaches its maximum value, dp/dt = 0, and Equation (3) becomes

(4) $$\begin{array}{c} \frac{f\omega }{S}[1+(\eta -\frac{1}{\delta })\frac{p_{m}}{f}]\frac{d\psi }{\mathrm{dt}}-\nu_{m}(1+\theta )p_{m}=0 \end{array}$$

where

(5) $$\begin{array}{c} \nu_{m}=\frac{SI_{k}}{\varphi m}(Z_{m}-Z_{0}) \end{array}$$

From Equations (4) and (5), the maximum chamber pressure can be obtained as below:

(6) $$\begin{array}{c} p_{m}=\frac{\frac{f\omega }{S}\cdot \frac{d\psi }{\mathrm{dt}}}{\frac{SI_{k}}{\varphi m}(Z_{m}-Z_{0})-(\eta -\frac{1}{\delta })\frac{\omega }{S}\frac{d\psi }{\mathrm{dt}}} \end{array}$$

The corresponding muzzle velocity is

(7) $$\begin{array}{c} \nu_{0}=\nu_{j}\{1-(\frac{\Lambda_{k}+1-\eta \Lambda }{\Lambda +1-\eta \Lambda }{)}^{\theta }[1-\frac{B\theta }{2}(1-Z_{0}{)}^{2}]\} \end{array}$$

where

(8) $$\begin{array}{c} \nu_{j}=\sqrt{\frac{2f\omega }{\theta \psi m}} \end{array}$$

(9) $$\begin{array}{c} B=\frac{S^{2}I_{k}^{2}}{f\omega \varphi m} \end{array}$$

Λ is the volume of the propellant.

Equation (6) and Equation (7) reveal that the values of p_m_ and v_0_ depend on the artillery structure and the propellant properties. The parameters f, ω, S, m and φ are determined by the artillery and the propellant charge and are independent of temperature. Only dψ/dt varies with temperature. It can be seen that the effect of temperature on chamber pressure and muzzle velocity comes from the effect of temperature on the propellant’s burning rate [34,35]. When temperature increases, the propellant’s burning rate and the values of pm and v_0_ increase significantly, leading to a higher temperature sensitivity coefficient of the weapons.

The following equation is obtained:

(10) $$\begin{array}{c} \frac{d\psi }{dt}=\frac{\sigma \chi }{e_{1}}\frac{de}{dt} \end{array}$$

On the right-hand side of this equation, σ is the relative burning surface area, e_1_ is half the web thickness of the propellant, de/dt refers to the web thickness varying with time, and χ is the characteristic shape parameter of the propellant. Except for de/dt, these parameters are basically independent of temperature. It can be derived that the temperature sensitivity coefficient of the propellant charge is generated not only by the temperature effect on the burning rate of the propellant but also by the change in dψ/dt. To maintain a nearly constant dψ/dt at different temperatures, it is necessary to keep de/dt unchanged or compensate for the loss of the burning rate caused by de/dt by changing the burning surface area at different temperatures. However, it is difficult to effectively keep the initial dψ/dt constant at different temperatures, so changing the burning surface area at various temperatures to compensate for the loss of the burning rate becomes the first choice. de/dt becomes larger and smaller with temperature rise and fall, so the burning surface area of the propellant should be adjustable at different temperatures to keep dψ/dt constant.

## 3. Experiment

### 3.1. Materials

Nitrocellulose was purchased from Sichuan Nitrocell Co., Ltd. (Luzhou, China), with a nitrogen content of 12.6%. RDX came from Gansu Yinguang Chemical Industry Group Co., Ltd. (Baiyin, China) and has an average diameter of 7.6 μm. Acetone and ethanol were from Nanjing Chemical Reagent Co., Ltd. (Nanjing, China) and are analytical-grade.

### 3.2. Preparation

**Figure 1:**
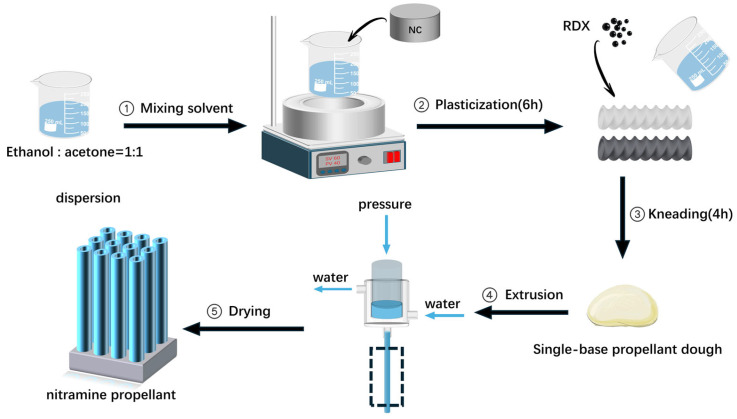
The process of the preparation of the sample propellant.

A mixed solvent of ethanol and acetone with a ratio of 1:1 was used to plasticize nitrocellulose at room temperature. After 6 h, the plasticized nitrocellulose was put into a kneader (1085, Shaanxi Zhongcheng Power Machinery Co., Ltd., Shaanxi, China) and kneaded for 4 h at 35 °C to prepare single-base propellant dough. For the modified single-base propellant, 5 wt% of ultra-fine RDX particles was added during the kneading process in several batches. Then, the dough was put into an extruder (1t, Hefei Haide CNC Hydraulic Equipment Co., Ltd., Hefei, China) and extruded to prepare a tubular single-base propellant with a dense structure and a 1.2 mm web thickness. The small size of RDX particles is beneficial to reduce the loss of the mechanical properties of the propellant. Figure 1 shows the detailed preparation process. Table 1 shows the composition of the modified and unmodified propellant samples.

### 3.3. TMA Test

**Figure 2:**
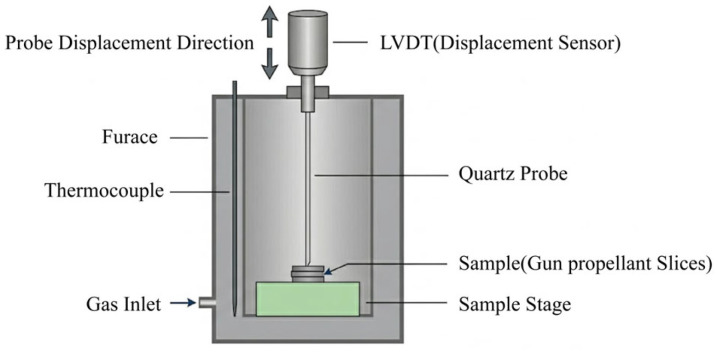
Simple schematic diagram of TMA device.

Temperature–deformation curves of the single-base propellant and the modified single-base propellant were recorded by a thermal mechanical analysis machine (TMA, TMA/SDTA841e, METTLER TOREDO, Greifensee, Switzerland). The thermal expansion rates of these samples were then derived from the obtained curves. These tests were carried out in the temperature range of −50 °C to 50 °C, with a heating rate of 5 °C per minute and a N2 protective atmosphere with a flow rate of 20 mL per minute. A schematic diagram of the device is shown in Figure 2:

### 3.4. SEM Observation

**Figure 3:**
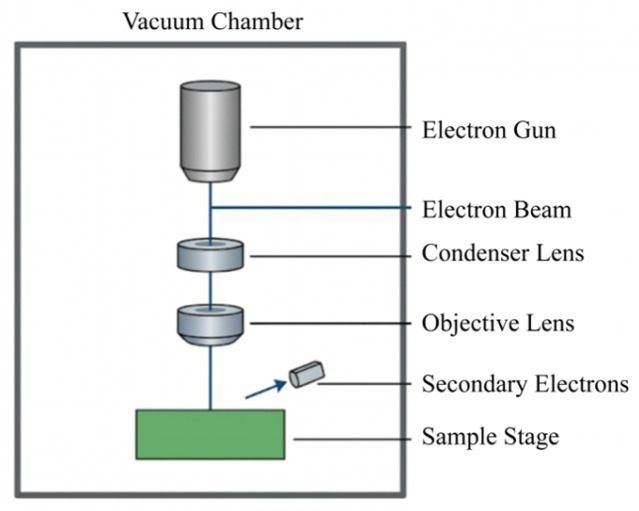
Simple schematic diagram of SEM.

In a high-vacuum environment (pressure not higher than 2600 Pa), the cross-sections of the single-base propellant and the modified single-base propellant were observed by a scanning electron microscope (SEM, Quanta250, FEI, Hillsboro, OR, USA). The samples were prepared according to SEM standard procedures and subjected to brittle fracture testing at 20 °C. A schematic diagram of the device is shown in Figure 3:

### 3.5. 3D Video Microscope

3D video microscopes (KH-1000 Hirox, Tokyo, Japan) have revolutionized microscopic observation by moving beyond the limited viewing area of eyepieces, enabling a more intuitive observation of specimens via displays. The microstructure of single-base propellants and modified single-base propellants with varying amounts of RDX, at both high and low temperatures, was observed under a 3D video scanning electron microscope at identical magnification levels.

### 3.6. Closed Bomb Test

The burning properties of the single-base propellant and the modified single-base propellant were tested by a closed bomb test at a temperature of −40 °C, 20 °C and 50 °C. The charge weight is 20 g. The propellant samples were cut into 40 mm long pieces and stored at −40 °C, 20 °C and 50 °C for 48 h before testing. The volume of the closed bomb is 98.15 mL. The chamber pressure was measured using a high-frequency pressure transducer with a sampling rate of 100 kHz.

Ignition was achieved using a standard 1.0 g 2# nitrocellulose igniter via an electric bridge wire [36]. Ignition pressure was included in the recorded p-t curves without separate correction, as its contribution was negligible compared to the main charge. Consistent ignition conditions were maintained across all tests to minimize systematic influence on the comparative analysis.

All experiments were conducted in triplicate under identical conditions to ensure reproducibility and minimize systematic influence on the comparative analysis.

## 4. Results and Discussion

### 4.1. Thermal Expansion Coefficient

The measured thermal expansion coefficients of the unmodified single-base propellant in different temperature ranges in the TMA test are listed in Table 2. The thermal expansion coefficient of RDX is 1.910 × 10^−4^ K^−1^ [37]. Compared with the measured data of the single-base propellant, which plays the role of the matrix in the modified single-base propellant listed in Table 2, the results indicate that the thermal expansion coefficient of RDX is much larger than that of the single-base propellant at any temperature. For modified single-base propellants, the coefficient of thermal expansion increases significantly upon the addition of RDX, due to the much higher thermal expansion coefficient of RDX compared to the nitrocellulose matrix. Moreover, the variation in this coefficient across different temperatures is noticeably reduced. It can be derived that the modified single-base propellant has less temperature sensitivity than the unmodified propellant. Due to the significant difference in the coefficients of thermal expansion between the single-base propellant matrix and RDX particles, the RDX particles undergo more pronounced shrinkage than the matrix under low-temperature conditions. This leads to the formation of microcracks at the adhesive interface between the dispersed phase and the continuous phase. These microcracks consequently increase the combustion surface area of the propellant. At high temperature, although RDX undergoes thermal expansion, the propellant matrix becomes softer and more flexible, partially offsetting the inflation gap, so the number of cracks induced will not be as large as that at low temperature, which implies that the burning area may remain the same as that at room temperature. This characteristic of forming microcracks at low temperatures and closing interfaces at high temperatures enables the self-regulation of the burning surface, thereby reducing the temperature sensitivity of combustion.

### 4.2. Internal Physical Structure

#### 4.2.1. SEM

**Figure 4:**
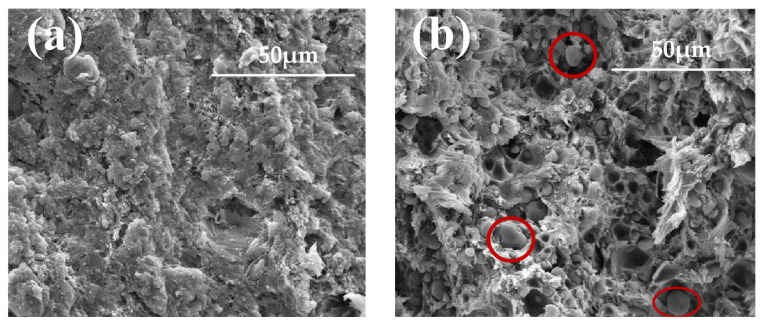
SEM images of internal structure of (**a**) single-base propellant and (**b**) modified single-base propellant.

Figure 4a,b displays the SEM images of the single-base and modified single-base propellants, respectively. From Figure 4a, it can be seen that the single-base propellant presents a dense, smooth and continuous phase. In Figure 4b, RDX particles are uniformly dispersed within the propellant matrix but exhibit weak adhesion to the single-base propellant matrix. A distinct phase interface can be clearly identified between RDX particles and the single-base propellant matrix, and several interfacial pores are also visible, which are formed by the shedding of RDX particles. Based on the parallel layer hypothesis for propellant combustion, this rougher interface surface indicates that the burning surface area of the modified single-base propellant is larger than that of the unmodified single-base propellant without RDX particles, thereby leading to a higher burning rate.

It is also seen from Figure 4b that more RDX particles are attached to the propellant substrate. This adhesion varies with temperature. In addition to the cumulative difference in thermal expansion, the adhesion is also affected by the tensile strength and viscosity of the matrix. As a polymer material, the propellant matrix gradually softens with increasing temperature, accompanied by improved tensile strength and viscosity. Because of the larger expansion of RDX particles (as shown by the red circle in Figure 4b), they press against the matrix, and this may result in less distance between them. This means that more RDX particles will be pressed against the interface at higher temperatures than at lower temperatures, resulting in a smaller burning surface area. As a result, the modified single-base propellant containing RDX particles has a burning surface area that varies with temperature, and this varying burning surface area will compensate to some extent for the reduction in the burning rate caused by temperature variation. This helps in achieving a low temperature sensitivity coefficient.

#### 4.2.2. 3D Video Microscope

The observation of the 3D video micrographs of the modified single-base propellant reveals that it exhibits a uniform and transparent state at elevated temperatures. However, at low temperatures, numerous microcracks generated by internal stress become apparent. These microcracks result from the contraction of the polymer material as temperature decreases. As indicated by TMA tests, RDX possesses higher temperature sensitivity than the single-base propellant. The microcracks can be attributed to the difference between RDX particles and the single-base propellant matrix. During cooling, their asynchronous contraction increases the number of cracks. Conversely, at high temperatures, both RDX and the propellant matrix expand, which reduces or even eliminates the cracks at their interfaces. Consequently, bonding strength at the two-phase interface between RDX particles and the single-base propellant matrix varies with temperature, demonstrating distinct interfacial characteristics.

**Figure 5:**
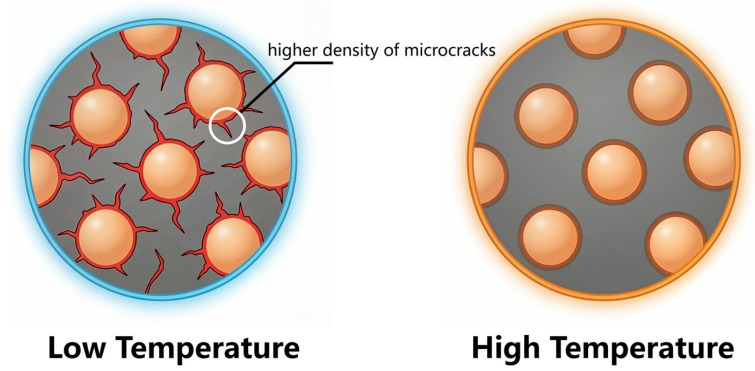
Schematic diagram of modified propellant structure under high and low temperatures.

The modified single-base propellant exhibits temperature-dependent microstructural evolution, characterized by a significantly higher surface crack density at low temperatures compared to high-temperature conditions. This disparity, which is markedly more pronounced in the modified variant than in its unmodified counterpart, originates from the shifting interfacial bonding strength between the nitrocellulose matrix and RDX particles. At low temperatures, the formation of these extensive microcracks provides a “surface area compensation” effect, effectively offsetting the inherent reduction in the burning rate. Conversely, the suppression of crack formation at high temperatures modulates the burning surface, preventing excessive gas generation [8]. This autonomous self-regulation mechanism helps maintain a stable gas generation rate across a wide temperature range, thereby fulfilling the objective of reduced temperature sensitivity. A schematic diagram is presented in Figure 5:

### 4.3. Combustion Performance

#### 4.3.1. Pressure–Time (p-t) Curves

**Figure 6:**
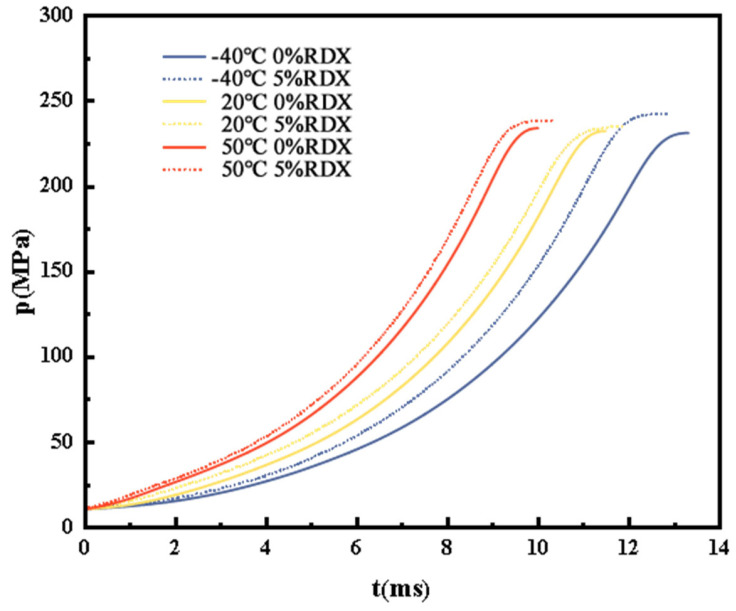
p-t curves of single-base propellant before and after modification at different temperatures.

Figure 6 shows the comparison results of the measured pressure–time history of the single-base propellant before and after modification at high, low and room temperature (50 °C, −40 °C and 20 °C, respectively).

As shown in Figure 6, the pressure–time curves of the modified single-base propellant are generally consistent with those of the unmodified propellant, indicating that the incorporation of ultra-fine RDX particles does not fundamentally alter the combustion mode under closed bomb conditions. The experimental results show that, at all tested temperatures, the modified propellant consistently exhibits a higher maximum chamber pressure (p_m_) than the unmodified propellant. Specifically, at 20 °C, 50 °C, and −40 °C, the p_m_ values of the unmodified propellant are 232.41 MPa, 234.10 MPa, and 231.27 MPa, respectively, while those of the modified propellant increase to 235.25 MPa, 238.84 MPa, and 242.64 MPa, respectively. This relative increase is particularly pronounced at low temperatures, suggesting that the addition of RDX helps compensate for the reduction in the reaction rate typically observed under such conditions.

Furthermore, compared with the unmodified propellant, the modified single-base propellant exhibits a shorter combustion duration at low temperatures but a longer combustion duration at high temperatures. This phenomenon is mainly ascribed to the weaker interfacial bonding between RDX particles and the propellant matrix at low temperatures. The resulting microcracks increase the burning surface area, thereby shortening the combustion time. Conversely, at high temperatures, the thermal expansion of both RDX particles and the matrix gradually reduces or even closes these cracks, leading to a decreased burning surface area and thus a prolonged combustion duration. This trend indicates that the modification alters the combustion dynamics in a temperature-dependent manner.

To further analyze combustion behavior, the evolution of the pressure–time curves at different combustion stages was examined. At low temperature (−40 °C), the modified propellant exhibits a rapid pressure rise during the later stage of combustion, contributing to a higher p_m_ that even exceeds the maximum pressure observed at 20 °C. This phenomenon suggests that the physical increase in the burning surface area compensates for the temperature-induced reduction in the chemical reaction rate and even enhances the pressure output to some extent.

Inevitable heat loss occurs during closed bomb experiments, generally accounting for approximately 5% of the total heat, which is considered negligible under practical operating conditions [32]. The interior ballistic method used in this study does not account for heat loss. However, our primary focus lies in the qualitative comparative analysis of the changes in propellant combustion behavior before and after modification, rather than precise quantitative calculations. The current experimental results sufficiently support the conclusions drawn. In practice, all experiments were conducted under identical conditions, which largely avoids any influence of heat loss on the experimental results.

#### 4.3.2. Γ-Ψ and L-B Curves

To further explore burning performance, Γ-Ψ curves were derived from the closed bomb test results. Γ is the vivacity of the propellant gas generated and describes the gas generation per unit pressure, which is determined by the following equation [32]:

(11) $$\begin{array}{c} \Gamma =\frac{1}{p}\frac{d\psi }{\mathrm{dt}}=\frac{\chi a}{\delta_{1}}\sigma p^{n-1} \end{array}$$

**Figure 7:**
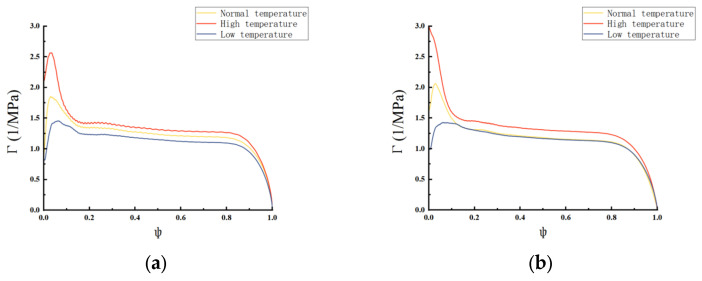
Γ-Ψ curves of (**a**) single-base propellant and (**b**) modified single-base propellant.

Ψ is the proportion of burnt propellant and ranges from 0 to 1. The Γ-Ψ curve reflects the relationship between the gas vivacity and weight percentage of the propellant burnt. The Γ-Ψ curves of the single-base propellant before and after modification at different temperatures are presented in Figure 7.

Figure 7a presents the Γ-Ψ curves of the single-base propellants. Under the same gas generation volume, the gas generation rate of the propellant increases with temperature, indicating that dΨ/dt varies with temperature. As discussed in Section 2, to achieve a low temperature sensitivity coefficient, dΨ/dt should be kept as constant as possible over the range of test temperatures.

For the unmodified propellant, the Γ-Ψ curves are clearly separated over the entire tested temperature range, indicating a strong temperature dependence of the gas generation rate. As temperature increases, the curves shift upward, reflecting the increase in the burning rate and gas generation volume with temperature.

In contrast, the differences among the curves of the modified propellant at different temperatures are significantly reduced. As shown in Figure 7b, when Ψ exceeds 0.15 for the modified single-base propellant, the Γ-Ψ curves at low temperature and room temperature nearly overlap, indicating that the temperature dependence of the gas generation process is effectively suppressed during the middle and late stages of combustion. This observation suggests that the modified propellant exhibits a lower temperature sensitivity coefficient.

**Figure 8:**
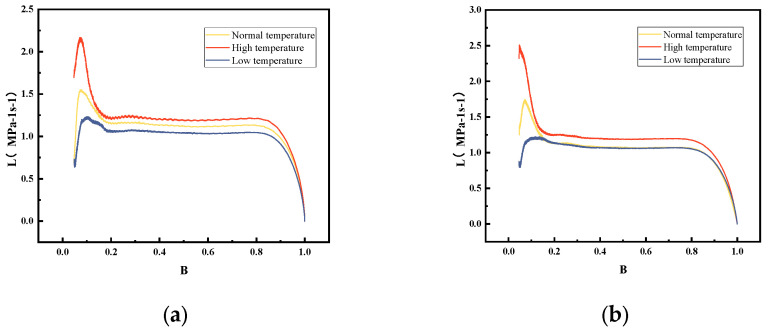
L-B curves of (**a**) unmodified single-base propellant and (**b**) modified single-base propellant at different temperatures.

This result is further supported by the L-B curves presented in Figure 8.

According to STANAG 4115, L is the vivacity of the propellant defined by the following equation [38]:

(12) $$\begin{array}{c} L=\frac{1}{p\cdot p_{m}}\cdot \frac{\mathrm{dp}}{\mathrm{dt}} \end{array}$$

B is the ratio of pressure to maximum pressure (p/p_m_).

The L-B curves reflect the variation in gas generation as a function of pressure ratio. When the relative pressure reaches 0.2, the L-B curves tend to converge and exhibit a stable trend without abrupt changes. This indicates that stable combustion is achieved in all tested cases, with no significant abnormal combustion phenomena observed. After modification, the L-B curves become more tightly clustered over the entire pressure range and display smoother profiles. This suggests that the combustion process becomes more stable and the propellant exhibits reduced sensitivity to temperature variations. Furthermore, the L values of the modified propellant are generally higher than those of the neat propellant at all tested temperatures, confirming that the incorporation of RDX effectively enhances the energy level of the single-base propellant.

This phenomenon can be attributed to the coupling effect of thermal expansion and the adhesion behavior of the propellant matrix. In the modified single-base propellant, the RDX particles are randomly distributed, forming a heterogeneous structure. In this structure, there are multiple interfaces, with potential gaps hiding in them. The number and scale of these gaps vary with temperature. As temperature decreases, the propellant matrix becomes harder, and the adhesive force decreases, which not only magnifies the difference between the RDX particles and the propellant matrix to a certain extent but also helps increase the number and scale of potential gaps. The result is that these potential gaps in the propellant at lower temperatures are not as few and unclear as they are at higher temperatures. This result suggests that a larger burning surface area at lower temperature compensates for the loss of the burning rate caused by the low temperature to some extent. In summary, through the self-regulating mechanism of increasing microcracks at low temperatures and reducing cracks at high temperatures, the modified propellant achieves the temperature-adaptive adjustment of the burning area. This enables it to maintain a relatively stable gas generation rate during the combustion process, which constitutes the core mechanism of its low temperature sensitivity performance.

This “self-regulation” mechanism, based on microstructural evolution, enables the modified propellant to achieve the dynamic adaptation of the burning surface area over a wide temperature range, thereby ensuring a nearly constant gas generation rate. The results demonstrate that the proposed modification strategy not only increases energy density but also effectively reduces the temperature sensitivity of combustion performance through a microscopic self-compensation mechanism, achieving an optimized balance between energy and sensitivity properties.

#### 4.3.3. u-p Curves

**Figure 9:**
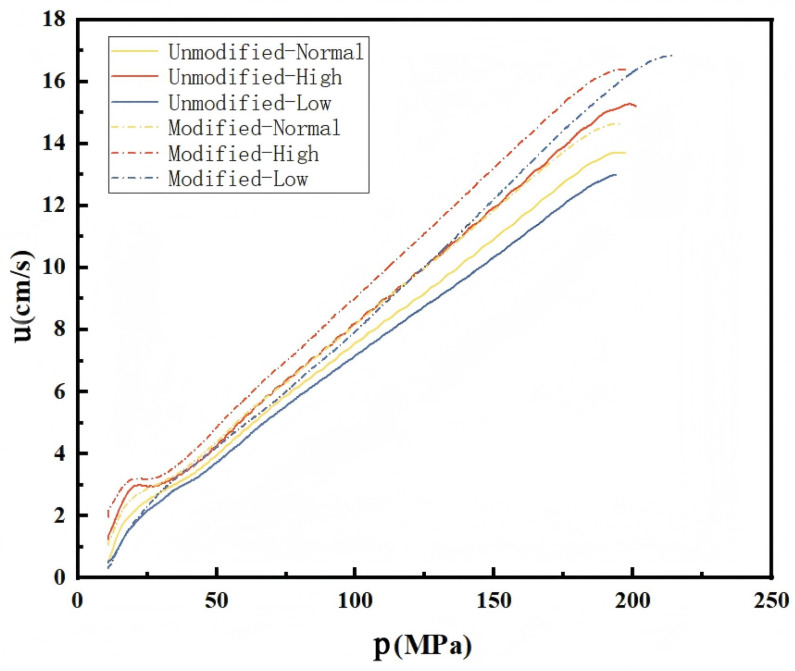
u-p curves of unmodified and modified single-base propellants at different temperatures.

The u-p curves reflect the relationship between the burning rate and the pressure of the propellant at different temperatures. The results are indicated in Figure 9. Combustion performance is relatively stable, with the burning rate increasing uniformly as pressure rises, showing no significant turning points on the curves. Under identical temperature and pressure conditions, the modified single-base propellant consistently exhibits a higher burning rate than the unmodified propellant.

At low temperatures, the burning rate of the modified propellant increases more rapidly. During the mid to late stages of combustion, it exceeds the burning rates observed under both normal and high-temperature conditions under the same pressure. This enhancement indicates a larger combustion surface area, which can be attributed to the significant debonding between the RDX particles and the nitrocellulose (NC) matrix, as well as the presence of more microcracks within the NC matrix itself. These structural features collectively contribute to the increased burning surface area.

Furthermore, compared to the unmodified single-base propellant, the u-p curves of the modified propellant at high and low temperatures lie closer to each other. This indicates that the burning rate of the modified propellant is less sensitive to temperature variations. It can therefore be inferred that the temperature coefficient of the modified single-base propellant is reduced.

#### 4.3.4. dp/dt-p Curves

**Figure 10:**
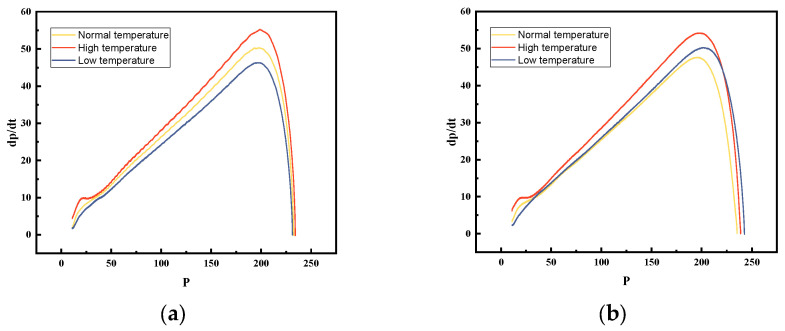
dp/dt-p curves of (**a**) unmodified single-base propellant and (**b**) modified single-base propellant at different temperatures.

The pressure rise rate of the unmodified single-base propellant varies significantly with temperature, while that of the modified propellant exhibits negligible temperature dependence. As shown in Figure 10, the three curves for the modified propellant at different temperatures are closely clustered and display broader peak regions. This indicates lower temperature sensitivity and more stable combustion, which aligns with the conclusions derived from the L-B curves.

At low temperatures, the modified propellant displays a markedly higher pressure rise rate and peak value, with its performance curve closely matching that at ambient temperature. This supports that the addition of RDX significantly counteracts the adverse effects of low temperature on combustion. On the one hand, the high energy density of RDX results in substantial heat release during the initial combustion stage, providing extra energy to sustain propellant combustion. On the other hand, low temperature widens the gap at the interface between RDX particles and the NC matrix, potentially causing cracks that allow combustion products to penetrate the pores of unburned propellant particles more easily [39], thereby expanding the burning surface area and increasing the burning rate.

#### 4.3.5. Burning Rate Temperature Coefficient and Pressure Exponent

The temperature dependence of the burning rate of the propellant can be generally expressed by the burning rate temperature sensitivity coefficient. This coefficient refers to the percentage of the change in the burning rate of the propellant within 1 °C close to the initial temperature under a certain pressure, which can be denoted by α [40,41]. When other impact factors are determined, the α measured by the closed bomb test can be used to characterize the temperature sensitivity coefficient of the propellant. Then, α can be expressed by Equation (13).

(13) $$\begin{array}{c} \alpha =\frac{r_{2}-r_{1}}{r_{1}(T_{2}-T_{1})} \end{array}$$

Here, r_1_ is the initial burning rate of the propellant at initial temperature T_1_, and r_2_ is the burning rate at temperature T_2_.

According to the differences in the temperature sensitivity coefficient of the burning rate across different test temperature ranges, the burning rate temperature coefficient can be defined as the derivative of the percentage of the change in the burning rate to the initial temperature under constant pressure. When the temperature change is sufficiently small, the following equation is obtained:

(14) $$\begin{array}{c} \alpha =\frac{\mathrm{dlnr}}{\mathrm{dT}} \end{array}$$

Integrating Equation (14), we obtain

(15) $$\begin{array}{c} ln\frac{r_{2}}{r_{1}}=\alpha (T_{2}-T_{1}) \end{array}$$

(16) $$\begin{array}{c} r_{2}=r_{1}e^{\alpha (T_{2}-T_{1})} \end{array}$$

Equation (15) expresses the relationship between the burning rate and the burning rate temperature sensitivity coefficient. From Equation (16), it can be derived that a smaller α leads to a more independent burning rate with respect to initial temperature. In other words, the propellant with a low burning rate temperature coefficient has a low temperature sensitivity coefficient under other certain conditions.

The pressure exponent n is a parameter that characterizes how pressure-sensitive a propellant’s burn rate is. Equation (17) is a widely used empirical formula for the burn rate.

The pressure exponent n is a parameter used to describe the sensitivity of the burning rate to pressure. The closer its value is to 1, the more sensitive combustion is to pressure perturbations. A typical empirical formula for the burning rate is as follows:

(17) $$\begin{array}{c} r=ap^{n\;} \end{array}$$

Here, r is the burning rate of the propellant, a is the burning rate coefficient, p is pressure, and n is the pressure exponent.

Burning rates at different pressures and temperatures can be obtained from the closed bomb test. The burning rate temperature sensitivity coefficient at a specific pressure and the pressure exponent at different temperatures can be calculated based on burning rate data at different temperatures. The calculated average burning rate; the burning rate temperature sensitivity coefficients at 50 MPa, 70 MPa, 100 MPa and 150 MPa; and the pressure exponent at 20 °C, −40 °C and 50 °C are listed in Table 3.

As shown in Table 3, the burning rates of the single-base propellant before and after modification increase with temperature. At the same temperature and pressure, the burning rate of the modified single-base propellant is higher than that of the unmodified propellant, while its burning rate temperature sensitivity coefficient decreases significantly. This indicates that the incorporation of RDX can enhance the energy level of the single-base propellant and further confirms its role in reducing temperature sensitivity. Additionally, the burning rate pressure exponent at different temperatures is also reduced, demonstrating improved combustion stability.

#### 4.3.6. Combustion Stability of Modified Single-Base Propellant

As mentioned earlier, the potential interfacial gaps between RDX particles and the single-base propellant matrix in the modified propellant are employed to reduce the temperature sensitivity coefficient. However, the safety of this strategy should be considered. By comparing the relative quickness (RQ) of the modified single-base propellant and the unmodified propellant, the combustion safety of the modified propellant can be evaluated.

The parameter RQ is defined as follows [42].

(18) $$\begin{array}{c} RQ=\frac{1}{n}\sum_{j=1}^{n}\frac{(\mathrm{dp}/\mathrm{dt}{)}_{\mathrm{reference}\;\mathrm{sample}}}{(\mathrm{dp}/\mathrm{dt}{)}_{\mathrm{test}\;\mathrm{sample}}} \end{array}$$

In Equation (18), the dp/dt values in the numerator and denominator are measured under the same specified instantaneous pressure, and the parameter n is the total number of measurements per test. Typically, n = 4.

The single-base propellant and the modified single-base propellant are taken as the unmodified sample and the test sample, respectively. The four groups of dp/dt values of these two samples are extracted from the measured p-t curves when p/p_m_ is 0.27, 0.4, 0.53 and 0.66. Here p_m_ refers to the maximum pressure in the p-t curve. Thus, the obtained RQ values at room, low and high temperature are 0.9639, 0.9892 and 0.9960, respectively. The RQ values remain consistently below unity and close to 1, indicating that the introduction of interfacial microcracks does not trigger abnormal combustion or hazardous pressure spikes. Instead, the microcracks only provide controlled compensation for the burning surface area, ensuring safe and stable combustion.

### 4.4. Mechanistic Analysis

The core mechanism underlying the reduced temperature sensitivity of RDX-modified single-base propellants lies in the significant mismatch in thermal expansion coefficients between RDX and the propellant matrix. This mismatch can enhance the performance of the base propellant. Through controlled processing conditions, ultra-fine RDX particles are uniformly dispersed within the nitrocellulose (NC) matrix while maintaining a certain degree of incompatibility, thereby forming a temperature-responsive phase interface.

At low temperatures, RDX particles contract more than the matrix, leading to interface debonding and the formation of microcracks. These structural changes increases the burning surface area, thereby accelerating the chemical reaction rate and compensating for the loss in the burning rate typically observed at low temperatures. At high temperatures, although the chemical reaction rate increases, the expansion of RDX particles tightens the interfacial bond, reducing the burning area and preventing an excessive increase in the burning rate. This dual mechanism is likely to contribute to more consistent combustion behavior across a wide temperature range.

By dynamically regulating the burning surface area through the temperature-dependent expansion and contraction of RDX particles, the modified single-base propellant achieves an approximate 70% reduction in the temperature sensitivity coefficient of the burning rate, accompanied by a lower pressure exponent and significantly improved combustion stability.

## 5. Conclusions

In order to obtain a single-base propellant with higher energy and a lower temperature sensitivity coefficient, a certain amount of RDX ultra-fine particles were added to the single-base propellant formula. The thermal expansion coefficient of RDX is markedly different from that of the single-base propellant matrix, and the adhesion force between the matrix and RDX varies with temperature. Therefore, in the heterogeneous single-base propellant, the property of the potential gaps in the interface changed with temperature, which was utilized to decrease the temperature sensitivity coefficient of the modified single propellant.

(1) SEM images show that the modified single-base propellant has a heterogeneous structure, and there are interfaces between RDX particles and the single-base propellant matrix. The mismatch in thermal expansion between the RDX particles and the propellant matrix, together with temperature-dependent interfacial adhesion, leads to a variable burning surface area in the modified single-base propellant, thereby reducing its temperature sensitivity coefficient.
(2) The results of the closed bomb test show that when the burned fraction Ψ is larger than 0.15, the gas generation rate of the modified single-base propellant at low temperature is approximately the same as that at room temperature. The modified single-base propellant exhibits a lower temperature sensitivity coefficient than the unmodified propellant.
(3) The combustion stability of the modified single-base propellant was evaluated using the RQ. The RQ values at room, low and high temperature are 0.9639, 0.9892 and 0.9960, respectively. All values are close to but less than 1, indicating that the modified single-base propellant can burn safely and stably.

## Figures and Tables

**Table 1:** The NC and RDX content of different samples.

Sample	Unmodified	Modified
NC (%)	100	95
RDX (%)	0	5

**Table 2:** TMA results of single-base and modified single-base propellants.

Temperature (°C)	Single-Base Propellant		Modified Single-Base Propellant	
	Thermal Expansion Coefficient (10^−4^ K^−1^)	Increment (%)	Thermal Expansion Coefficient (10^−4^ K^−1^)	Increment (%)
−30~0	0.141	−13.50	0.457	−3.38
0~20	0.163	-	0.473	-
20~50	0.150	−7.98	0.455	−3.81

**Table 3:** Calculated burning rate, burning rate temperature coefficient and burning rate pressure exponent of unmodified and modified single-base propellants.

Pressure (MPa)	Temperature (°C)	Burning Rate (cm/s)		Burning Rate Temperature Coefficient × 10^−4^/°C		Burning Rate Pressure Exponent	
		Single-Base Propellant	Modified Single-Base Propellant	Single-Base Propellant	Modified Single-Base Propellant	Single-Base Propellant	Modified Single-Base Propellant
50	20	3.96	4.47	10.60	2.86	20 °C 0.918	20 °C 0.888
	−40	3.72	4.39	10.60	2.86		
	50	4.25	4.86	10.60	2.86		
70	20	5.54	5.98	10.79	2.09		
	−40	5.21	5.90	10.79	2.09	−40 °C 0.928	−40 °C 0.897
	50	5.96	6.61	10.79	2.09		
100	20	7.59	8.17	9.60	1.88		
	−40	7.17	8.08	9.60	1.88		
	50	8.22	9.04	9.60	1.88	50 °C 0.936	50 °C 0.909
150	20	10.92	11.87	9.01	1.33		
	−40	10.36	11.78	9.01	1.33		
	50	11.95	13.23	9.01	1.33		

## Data Availability

The original contributions presented in this study are included in this article. Further inquiries can be directed to the corresponding authors.

## Abbreviations

The following abbreviations are used in this manuscript:

RDX	research department explosive
SEM	scanning electron microscope
TMA	thermal mechanical analysis
RQ	relative quickness
*f*	powder force
*φ*	coefficient of secondary works
2*e_1_*	web thickness
*Γ*	vivacity of propellant
*Ψ*	proportion of burnt propellant
*p*	pressure
*p_m_*	maximum pressure
*L*	vivacity of propellant
*B*	p/pm
*S*	cross-section area of muzzle
*ω*	mass of propellant
*l*	projectile travel
*l_0_*	length of chamber
*l_ψ_*	equivalent length of propellant gas
*v*	velocity
*Λ*	volume
*θ*	thermal parameter
*η*	covolume of propellant gas
*α*	temperature sensitivity coefficient
*δ*	density
*Δ*	loading density
*Z*	relative burning thickness of propellant
*χ*	characteristic shape parameter
*I_k_*	full pressure impulse
*T*	temperature
*t*	time
*m*	mass
*σ*	relative burning surface area
*a*	burning rate coefficient
*r*	burning rate
*n*	burning rate pressure exponent
